# Causal effects of genetically predicted endometriosis on breast cancer: a two-sample Mendelian randomization study

**DOI:** 10.1038/s41598-023-43999-7

**Published:** 2023-10-12

**Authors:** Shuixin Yan, Jiadi Li, Jiafeng Chen, Yan Chen, Yu Qiu, Yuxin Zhou, Weizhu Wu

**Affiliations:** 1 The Affiliated Lihuili Hospital, Ningbo University, Ningbo, Zhejiang China; 2 Health Science Center, Ningbo University, Ningbo, Zhejiang China

**Keywords:** Breast cancer, Cancer epidemiology, Cancer genetics, Cancer genomics, Cancer, Genetics

## Abstract

This study used a Mendelian randomization (MR) approach to investigate the causal relationship between genetically predicted endometriosis (EMS) and breast cancer risk. A total of 122,977 cases and 105,974 controls were included in the analysis, with gene-level summary data obtained from the Breast Cancer Association Consortium (BCAC). An inverse variance-weighting approach was applied to assess the causal relationship between EMS and breast cancer risk, and weighted median and MR-Egger regression methods were used to evaluate pleiotropy. Results showed a causal relationship between EMS and a decreased risk of overall breast cancer (odds ratio [OR] 0.95; 95% CI 0.90–0.99, p = 0.02). Furthermore, EMS was associated with a lower risk for estrogen receptor (ER)-positive breast cancer in a subgroup analysis based on immunohistochemistry type (OR 0.91; 95% CI 0.86–0.97, p = 0.005). However, there was no causal association between ER-negative breast cancer and survival (OR 1.00; 95% CI 0.94–1.06, p = 0.89). Pleiotropy was not observed. These findings provide evidence of a relationship between EMS and reduced breast cancer risk in invasive breast cancer overall and specific tissue types, and support the results of a previous observational study. Further research is needed to elucidate the mechanisms underlying this association.

## Introduction

In 2020, there were approximately 2.3 million new breast cancer cases worldwide, accounting for 11.7% of all new cancer cases. Women have a high mortality rate due to breast cancer (15/10,000)^1^. Depending on a woman’s advanced menopausal state and the status of the tumor receptors, breast cancer is a heterogeneous disease. The development of breast cancer is the result of a combination of internal and external factors. Some studies have identified certain risk factors for breast cancer, including age, family history, history of benign breast disease, history of estrogen use, lifestyle, obesity, and fertility^2^.

Endometriosis (EMS) is one of the most prevalent diseases in women of childbearing age, with a prevalence of 5–10%^3^. Numerous epidemiological and clinical studies have shown that EMS is estrogen-dependent. Estradiol (E2) has been shown to promote adhesion, invasion, proliferation, apoptosis inhibition, and inflammatory response maintenance in ectopic lesions. Elevated estrogen levels in ectopic lesions in patients with EMS suggest that local estrogen metabolism plays an important role in EMS development^4^. Additionally, extensive epidemiological data indicate that chronic estrogen exposure increases the likelihood of breast cancer^5^. Therefore, whether there is an association between EMS and breast cancer is a question of interest. Previous observational studies have yielded inconsistent results in EMS and breast cancer risk examination, with most showing implied increased risk (standardized incidence ratio, 1.3; 95% confidence interval [CI] 1.1–1.4)^6^. However, in the most recent largest study, international collaboration on breast cancer research reported a recommendation for a reduced risk of invasive breast cancer in women who self-report EMS^7^. The relationship between EMS and breast cancer has not been systematically studied because of potential biases such as confounding factors or reverse causality. Therefore, there is no conclusive evidence that EMS contributes to breast cancer progression. Additional investigations are needed to draw definitive conclusions regarding the causality and biology of these associations.

Mendelian randomization (MR) was used to overcome these limitations. This analytical method uses random genetic classification from parents to progeny to evaluate the association between EMS and breast cancer risk. When specific assumptions are met, this approach is largely independent of biases inherent in standard observational studies. A previous study has shown that EMS has a significant genetic component^8^, suggesting that MR may provide a way to examine the EMS-breast cancer relationship. Therefore, we sought to examine this association using information from recent genome-wide association studies (GWAS) on EMS and breast cancer.

This study aimed to investigate genetically predicted EMS with the risk of overall breast cancer and cancer by estrogen receptor status (estrogen receptor-positive [ER+] and estrogen receptor-negative [ER−]) using MR methodology.

## Materials and methods

### Mendelian randomization design

**Figure 1:**
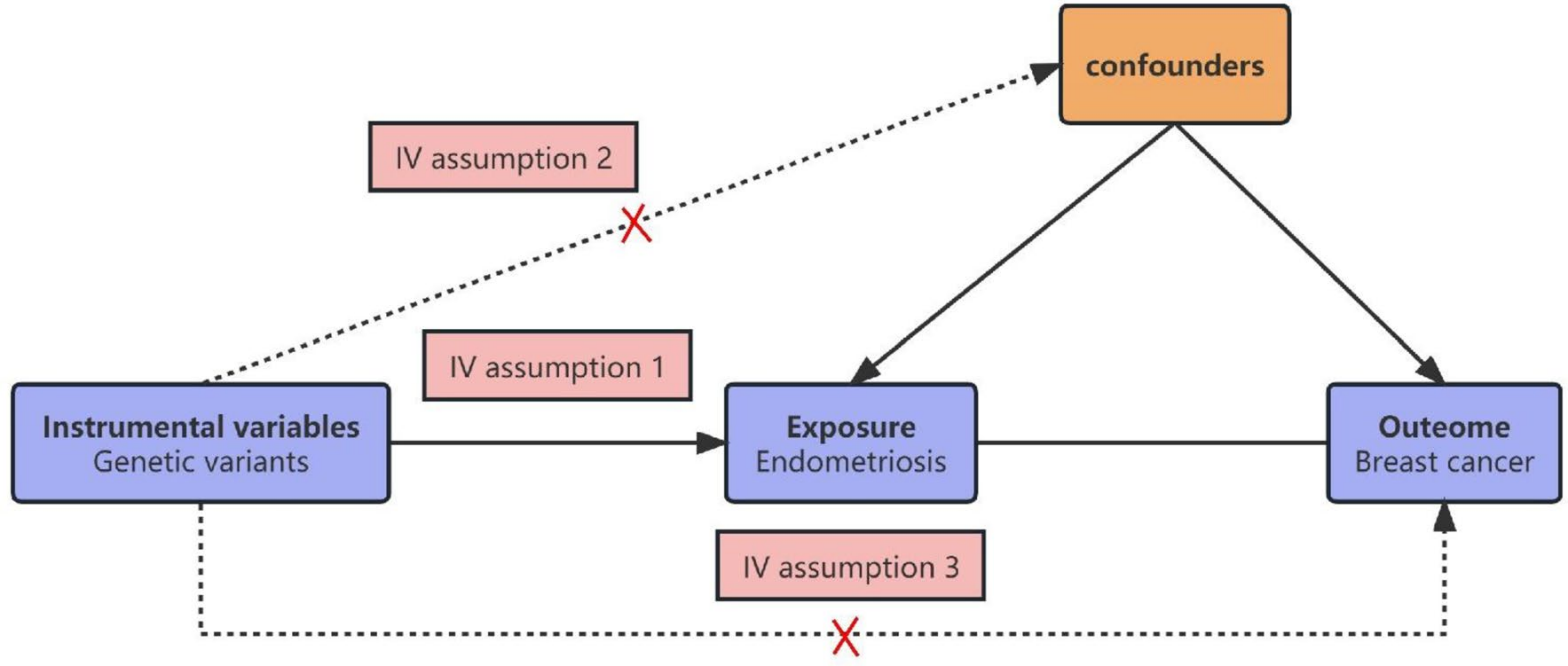
The design concept of this study. The accuracy of estimating causality using MR analyses is based on the following three assumptions: the instrumental variable (IV) associate with the exposure (IV assumption 1). This assumption can be satisfied by ensuring F statistic > 10 and that SNPs are selected using genome-wide significance levels (P < 5 × 10^–8^), (2) The IV is independent of combined influence of all confounders (IV assumption 2). For the same population and reference, we assess correlation of linkage disequilibrium between SNPs associated robustly with exposure and SNPs linked to possible known confounders. If the correlation coefficient is higer (i.e. r^2^ ≥ 0.5), the corresponding selected SNPs will be discarded. (3) The IV is independent of the outcome given the exposure and confounders (IV assumption 3), Horzontal pleiotroy, that IVs influence the outcome through alternative pathways other than the exposure could violate this assumption. It can be checked by using MR-Egger regression and MR-PRESSO method.

In this study, we used MR to explore the relationship between exposure and outcome. Among them, the inclusion of instrumental variables mainly follows the three principles of MR^10^. Firstly, we selected Instrumental variables (IVs) significant relationship with EMS (P < 5 × 10^−8^, r^2^ < 0.001, genetic distance = 10,000 KB, minor allele frequency > 0.01) at the genome-wide level. Secondly, to satisfy the independence of genetic variation and confounders, we searched in Catalog and PhenoScanner databases to ensure that each IV included was unrelated to known confounders. Finally, we calculated the F statistic to avoid the bias of weak IVs and confirmed that these results were not affected by weak IVs. The design concept of this study are shown in Fig. 1.

## Data sources

### Genetic instrument variants for exposure and outcome

EMS data were obtained from the FinnGen project (FinnGen), including 2953 EMS cases and 68,969 control participants. The samples of participants in our study for genetic analysis were obtained from the BCAC; a total of 122,977 cases and 105,974 controls were utilized, containing 38,197 ER+ cases and 21,468 ER− cases^9^. For the Breast Cancer Association Consortium dataset (BCAC), we recommend that the reader refer to the main GWAS manuscript and its Supplementary Material for more information on the consent protocols for the respective cohorts.

### Statistical analysis

Mendelian randomization (MR) methods were employed in this study. And three different analytical methods were used in the MR analysis to prevent this. Each analysis was based on a different horizontal multiplicity model. The benefit of comparing these three results is that the consistency of the three methods makes the results more credible. The main analysis was performed using an inverse variance-weighting (IVW) approach, which provided the most accurate estimates but assumed that all SNPs were valid IVs. MR-Egger approach is based on the assumption of InSIDE in order to perform a weighted linear regression of exposure results, but it is susceptible to IVs. Weighted median approach could significantly improve the detection ability of causal effects as well as reduce type I errors. MR-PRESSO is a variant of IVW that eliminates IVs and its causal estimates differ significantly from other IVs. It provides accurate analysis if horizontal pleiotropy occurs in less than 50% of IVs.

### Sensitivity analysis

We used different methods for assessing potential effects to ensure the third MR hypothesis that the IVs were independent of the outcome except for exposure^11,12^. Cochran Q statistics and MR-Egger regression were used to explain this study's heterogeneity and pleiotropy. Then, MRPRESSO, the leave-one-out analysis, and funnel plot were also used as additional multiplicity controls to global, outlier, and distortion tests.

All data were obtained from published studies approved by the institutional review boards, and informed consent was obtained from the participants of the original study. Therefore, no further sanctions were required. And all analyses were carried out using two-Sample MR and MRPRESSO packages in R software (Version 4.1.2, R Foundation). The OR value is mainly converted through beta exp(β). Significance levels were set at 0.05.

### Ethics statement

The GWAS data used in this study were public de-identified data. The ethics committee approved these data; therefore, there was no need for additional ethical approval.

### Consent to participate

Informed consent was obtained from all individual participants included in the study.

### Consent to publish

Authors are responsible for correctness of the statements provided in the manuscript. See also Authorship Principles. The Editor-in-Chief reserves the right to reject submissions that do not meet the guidelines described in this section.

### Accordance statement

It was confirmed that all protocols were approved by the Lihuili Hospital Committee of Ningbo University. Confirm that all methods are implemented in accordance with relevant guidelines and regulations.

## Results

### Instrumental variables for EMS

**Figure 2:**
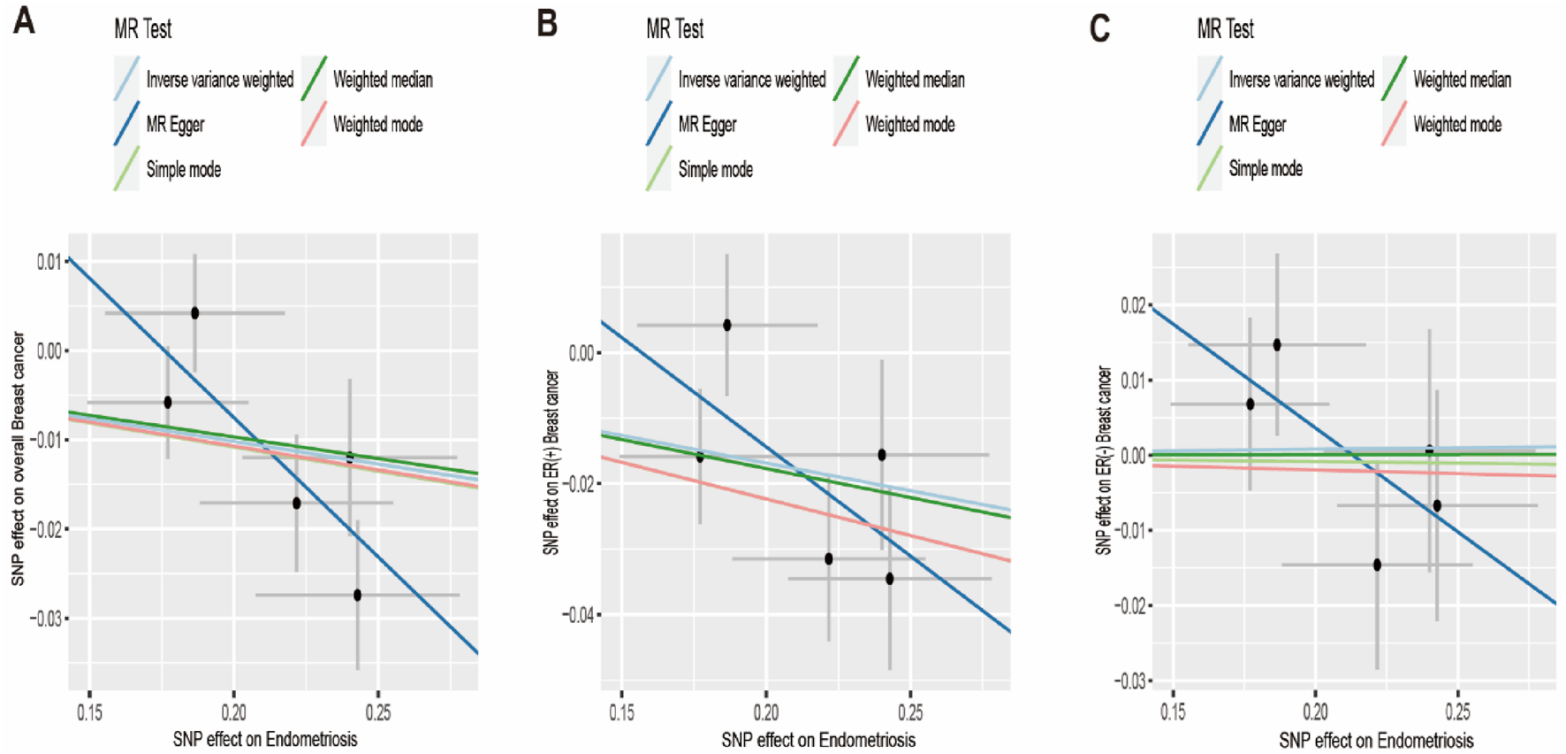
Scatter plot to visualize causal effect of EMS on overall breast cancer, ER(+)breast cancer and ER(−) breast cancer risk. The slope of the straight line indicates the magnitude of the causal association. IVW indicates inverse-variance weighted; and *MR* Mendelian randomization. *EMS* Endometriosis, *ER* Estrogen Receptor.

**Figure 3:**
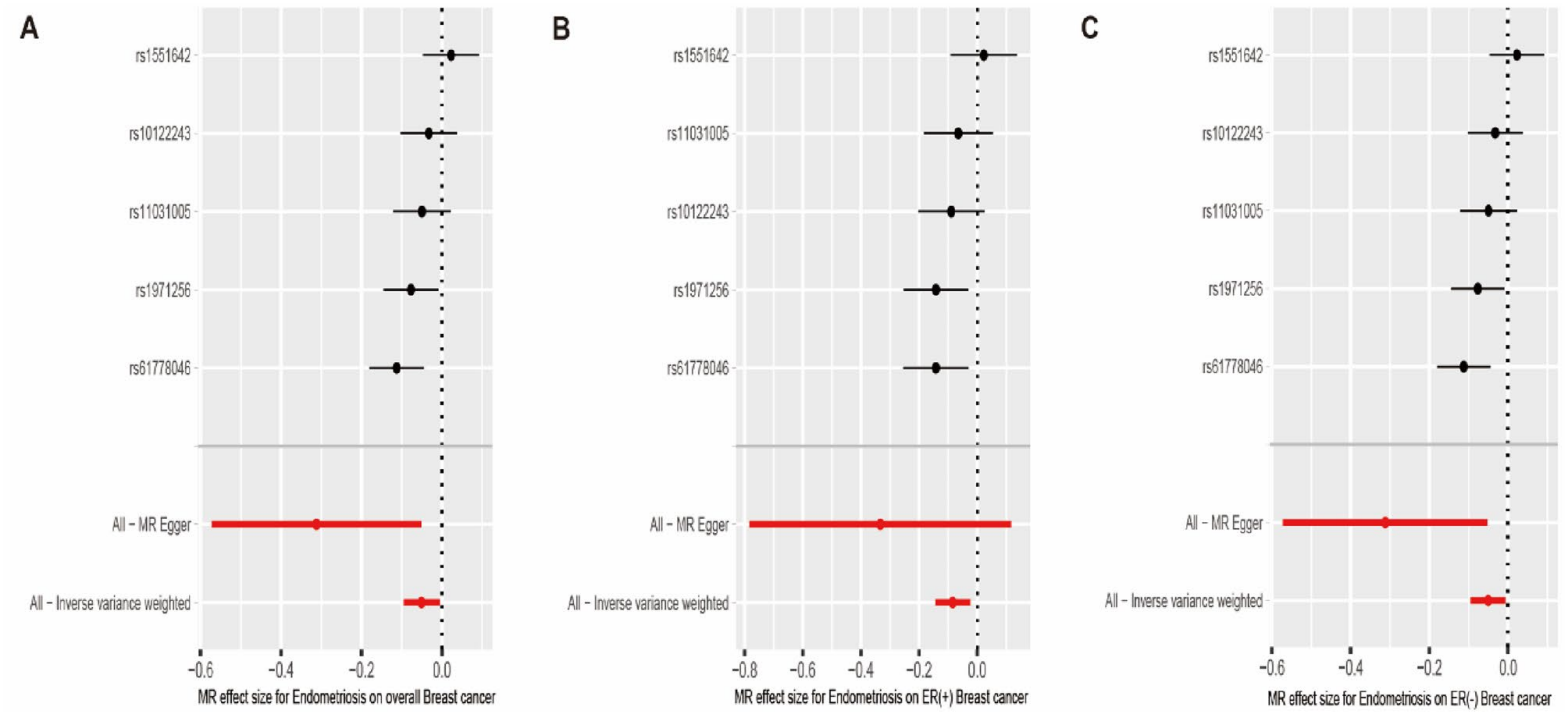
Forest plots showing beta (± standard error) and p-values of the single-SNP 2SMR analysis between endometriosis and overall breast cancer, ER(+)breast cancer and ER(−) breast cancer. *SNP* Single Nucleotide Polymorphism, *2-SMR* two-Sample Mendelian Randomization, *ER* Estrogen Receptor.

The SNPs’ signatures of the EMS are shown in Table 1. Finally, we selected five SNPs as the IVs. All genetic tools related to EMS were at a genome-wide significance level (p < 5 × 10^−8^, F > 10). Thus, none of the SNPs was susceptible to IVs. The causal effects of each genetic variant on breast cancer are shown in Figs. 2 and 3.

**Table 1:** List of genetic instruments for EMS and log odds ratios of osteoporosis risk by each instrumental SNPs (GWAS signifificance with p < 5 × 10^−8^ and linkage disequilibrium threshold with R^2^ < 0.001).

	SNP	Gene	Chr	EA	OA	EAF	β	SE	*p*	F
1	rs10122243	–	9	T	C	0.4116	0.1771	0.0279	2.26E−10	40
2	rs11031005	LOC105376607	11	C	T	0.1697	− 0.2401	0.0372	1.10E−10	41
3	rs1551642	–	4	C	T	0.2707	− 0.1865	0.0312	2.32E−09	36
4	rs1971256	CCDC170	6	C	T	0.2214	0.2217	0.0335	3.58E−11	44
5	rs61778046	CDC42-AS1	1	T	G	0.1903	0.2428	0.0353	5.83E−12	47

*EMS* Endometriosis, *SNP* Single Nucleotide Polymorphism, *GWAS* Genome-wide association studies, *EA* Effect allele, *OA* Other allele, *EAF* Effect allele frequency, *β* beta, *SE* Standard error.

### Mendelian randomization analyses for breast cancer

We evaluated the causal relationship between EMS levels and breast cancer using IVW, MR-Egger, and weighted median regression. Our findings suggest a reduced risk of breast cancer in patients with EMS (OR 0.95; 95% CI 0.90–0.99, p = 0.02). Subgroup analysis based on the type of immunohistochemistry showed a higher risk of ER+ breast cancer in patients with EMS (OR 0.91% CI 0.86–0.97, p = 0.005), whereas no significant correlation was observed between EMS and ER– breast cancer (OR 1.00; 95% CI 0.94–1.06, p = 0.89) (Table 2).

**Table 2:** Mendelian randomization estimates of the causality between genetically predicted EMS and breast cancer.

Outcome	IVW method		MR-egger		Weighted median method		Heterogeneity	Pleiotropy
	OR (95% CI)	p value	OR (95% CI)	p value	OR (95% CI)	p value		
Breast cancer overall	**0.95 (0.90, 0.99)**	**0.02**	0.73 (0.56, 0.94)	0.10	**0.95 (0.90, 0.99)**	**0.04**	**0.31**	**0.14**
ER-positive breast cancer	**0.91 (0.86, 0.97)**	**0.005**	0.71 (0.45, 1.12)	0.24	**0.91 (0.85, 0.98)**	**0.01**	**0.27**	**0.35**
ER-negative breast cancer	1.00 (0.94, 1.06)	0.89	0.75 (0.49, 1.17)	0.30	1.00 (0.92, 1.07)	0.99	0.69	0.29

Significant values are in bold.

*EMS* Endometriosis, *OR* Odds ratio, *ER* Estrogen Receptor, *IVW* inverse-variance weighted.

### Heterogeneity and pleiotropy analysis

**Figure 4:**
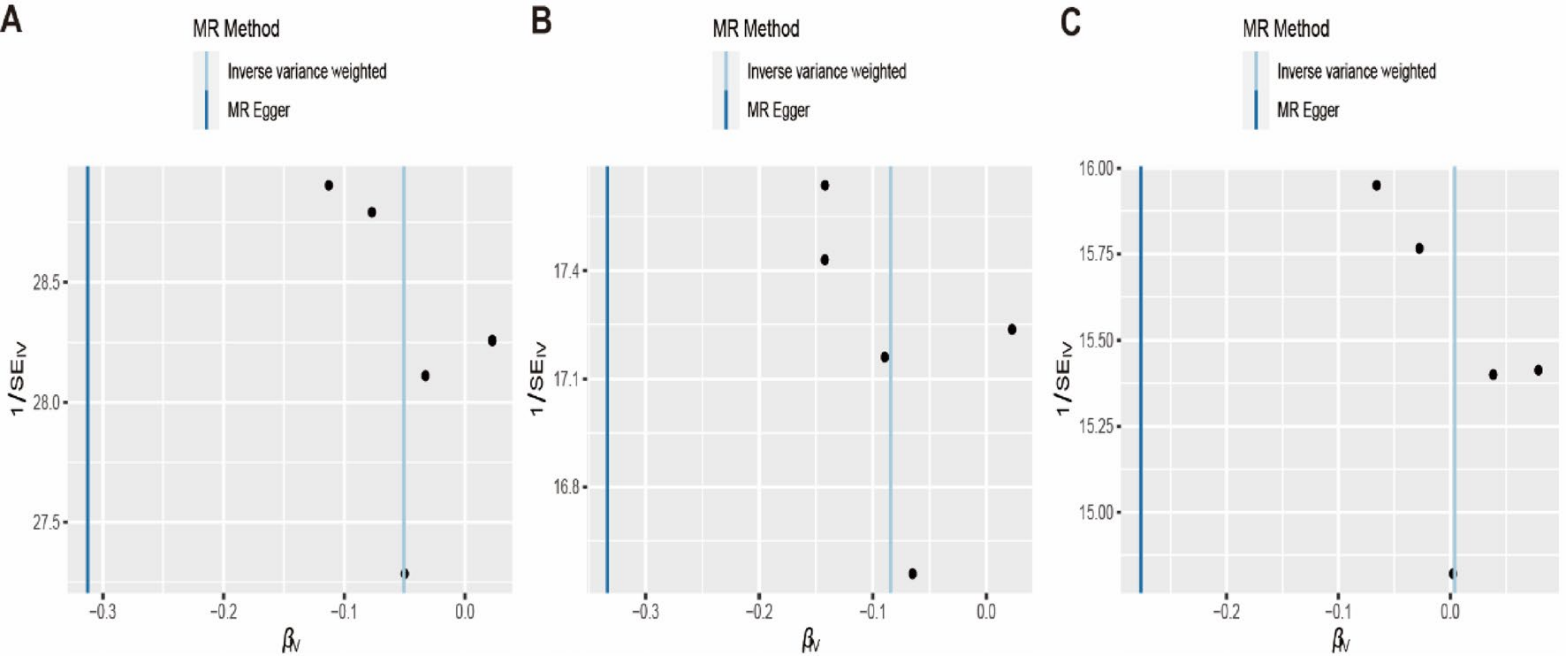
Funnel plots to visualize overall heterogeneity of MR estimates for the effect of EMS on breast cancer. IVW indicates inverse-variance weighted; and *MR* Mendelian randomization, *EMS* endometriosis, *IVW* Inverse variance-weighting.

**Figure 5:**
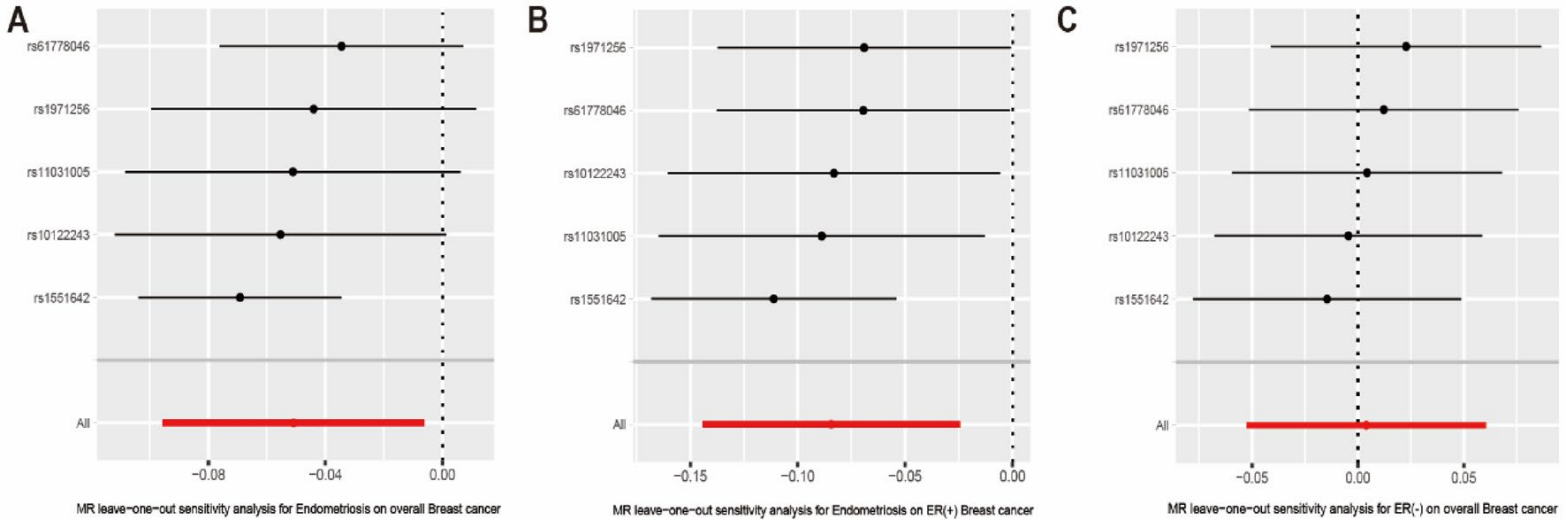
Leave-one-out plot to visualize causal effect of EMS on overall breast cancer, ER(+)breast cancer and ER(−) breast cancer risk. *EMS* Endometriosis, *ER* Estrogen Receptor.

Funnel plots can plot a single Wald ratio per SNP to display the directional level pleiotropy of the IVs. Nevertheless, the small number of IVs included makes it difficult to test for horizontal pleiotropy using funnel plots. The causal effect of the funnel plot was approximately symmetrical (Fig. 4). Leave-one-out analyses were performed to investigate whether estimates from IVW analyses were biased or dictated by individual SNPs, during meta-analyses that were conducted based on rerun IVW results for the remaining SNPs after omitting one SNP per succession. After removing each SNP, we performed MR analysis again systematically for the remaining SNPs. The results were consistent, indicating a significant causal relationship between the calculated results for all the SNPs (Fig. 5).

In MR analysis, the second hypothesis is that SNPs inject results only by modifying the exposure of interest, without other confounding pathways. Directional multidirectionality was examined to obtain the intercept and p-value using MR-Egger regression. No horizontal pleiotropy was observed in the intercept of the MR-Egger regression (p > 0.05), further indicating that pleiotropy did not bias the causal effect. Furthermore, in the published GWAS, there was no evidence that the included EMS-associated SNPs were significantly associated with any phenotype except EMS, which indicates that the assumptions of the third MR were not violated.

## Discussion

This study evaluated the causal relationship between EMS and breast cancer using a Mendelian randomization approach. We found that each SD increase in EMS predicted a 5.0% decrease in breast cancer risk. The inference still held in the subtype analysis; in patients with ER + breast cancer, each SD increase in EMS predicted a decrease in the risk of breast cancer by 8.1%; however, no causal relationship was observed in ER– patients. In addition, to exclude the possibility that this causal inference received confounding factors, potential confounding factors, such as obesity and smoking, were analyzed. Consequently, we considered that confounding factors did not confound the causal inference.

Despite being benign, EMS also has the characteristics of a malignant tumor^13^. Many studies have investigated the relationship between EMS and breast cancer; however, no consistent conclusion has been reached. Some studies have confirmed that EMS increases the risk of breast cancer^14–16^. Few studies considered EMS protective against breast cancer^7,17,18^. Other studies have argued that there is no relationship between EMS and breast cancer^19,20^. The possible selection and detection biases in these studies could significantly hamper the data assessment. For example, the selection of women undergoing endometrial surgery in some studies has led to potential selection bias. Additionally, the choice to use a discharge diagnosis of EMS contributed to detection bias, as only severe cases were included in the study. Selection and detection bias excluded many patients with cancer from the study, which might have led to an inaccurate assessment of EMS developing into breast cancer. Simultaneously, some studies used a self-reported format (telephone callbacks and questionnaires), which may have led to recall bias.

The fundamental pathological change in EMS is not proliferation of epithelial cells, but an increase in inflammation and cell survival resulting from apoptosis or diminished differentiation^21^. Inflammatory responses due to overproduction of reactive oxygen species are also present in breast cancer patients^22^. One study reported that women who underwent bilateral oophorectomy with hysterectomy because of EMS experienced a 58% decrease in breast cancer risk. This might be owing to an early interruption of the inflammatory process that could lead to breast cancer risk^23^.

The function of estrogen is mediated by two estrogen receptors (ERα and ERβ). Studies on EMS have shown increased levels of ERβ and decreased levels of ERα in endometriotic tissue compared to that in the normal endometrium^24,25^. Reduced methylation of CpG islands in the ERβ gene promoter leads to increased expression levels in endometrial stromal cells, whereas hypermethylation silences ERβ expression. ERβ in endometrial stromal cells dominated the ERα promoter and deregulated its activity, which facilitated the suppression of ERα levels, leading to an altered ERβ:ERα ratio in tissues^26^. It was shown that in breast cancer, the expression of ERβ was decreased compared to that in normal tissues, suggesting a protective effect of ERβ against ERα-induced overproliferation^27^. Women diagnosed with EMS in the postmenopausal period have an increased risk of breast cancer. It is possible that a higher Erβ level in women with EMS leads to a better prognosis. Our study also found a protective effect of EMS against ER+ breast cancer but not against ER– breast cancer.

The consensus among clinicians and researchers is that estrogen increases the risk of laparoscopically visible EMS and associated pelvic pain, and that targeting cyclooxygenase-2 in the estrogen biosynthesis pathway and the prostaglandin pathway decreases or eliminates laparoscopically visible EMS and pelvic pain^28,29^. Women younger than 40 years of age had a reduced risk of developing breast cancer when diagnosed with EMS compared with women older than 40 years. The reduced risk among younger women may be because of their exposure to anti-estrogenic drugs. The increased risk in menopausal women may be owing to a common risk factor between postmenopausal EMS and breast cancer^15^. Thus, the use of anti-estrogen drugs in patients with EMS might reduce the risk of breast cancer.

It has been shown that when comparing DNA repair capacity (DRC) in both EMS and breast cancer, women with EMS were 10% less likely to have low DRC compared to women without EMS, and those diagnosed with EMS at 38 years of age were 40% less likely to have low DRC. A low DRC is an important risk factor for breast cancer, as reported in the literature and in the same group of women. The mechanisms associated with the protection of breast cancer by EMS and the positive association between EMS and DRC remain largely unknown. Some drugs may also alter DRC, indirectly affecting the risk of breast cancer. As more is known about the molecular similarities between EMS and breast cancer, this knowledge should provide more effective strategies to prevent and treat both conditions^17^.

The standardized incidence of in situ breast cancer increased in the 40- to 59-year-old age group; however, patients with EMS aged 40 years or older might undergo breast imaging more frequently than the general population, which could potentially increase the detection and prompt treatment of in situ cancer and thus reduce breast cancer risk^30^.

This study has several strengths. The MR analysis was used for the first time to evaluate the causal association between EMS and breast cancer. Since genetic mutations are inherited from the parental generation, they do not receive external environmental influences and are therefore not subject to reverse causality, which is often present in observational studies. Additionally, we excluded the influence of potential confounding factors. Moreover, we derived these data from published GWAS data. The GWAS data contained 2,953 cases and 68,969 controls, which is a large sample size that made our study more convincing.

This study also has some limitations. First, our study indicated that EMS reduced the risk of breast cancer and provided more insight into the clinical disease. However, this has limitations in clinical application since one cannot make people with a high risk of breast cancer develop EMS, which would be difficult to achieve and unethical. Second, only a weak protective effect was observed. Third, our study population was limited to European ancestors, and further studies are needed to determine whether this is feasible in other populations.

In conclusion, these findings provide evidence of an association between EMS and reduced breast cancer risk, with the strongest correlation observed in ER+ breast cancer. These results are in agreement with those of our previous analysis of a large pooled epidemiological study. Further studies are needed to understand the mechanisms underlying this association.

## Data Availability

The original contributions presented in the study are included in the article, further inquiries can be directed to the corresponding authors.
